# A quantitative exploration of Queer-spectrum students’ experiences in introductory undergraduate mathematics courses

**DOI:** 10.1371/journal.pone.0275325

**Published:** 2022-10-07

**Authors:** Matthew Voigt

**Affiliations:** Department of Engineering and Science Education, Clemson University, Clemson, South Carolina, United States of America; Williams College, UNITED STATES

## Abstract

Research into the experiences of students in mathematics with a marginalized sexual identity has remained largely underexplored, relegating these experiences and individuals to remain invisible. In this study, we leverage Queer Theory to address this gap in research by reporting on the experiences of Queer-spectrum undergraduate students (*n* = 2,454) enrolled in introductory mathematics courses (Precalculus, Calculus 1, and Calculus 2) across the United States. Drawing on student data (*n* = 24,327) from the Student Post-Secondary Instructional Practices Survey, we examine reported outcomes of math learning experiences and access to learning environments. Overall, within introductory math courses, sexual identity had a significant relationship with accessing learning environments and mathematical learning experiences. Asexual students were among those that reported higher levels of interactions with peers and instructors, a greater sense of community & participation, positive math affect, and were more likely to access learning environments external to the course yet anticipated receiving a lower course grade when compared to straight students. Bisexual students reported lower levels of interactions with instructors, a diminished sense of community & participation, math engagement, positive math affect, and were less likely to access learning environments compared to straight students. These findings suggest that Queer-spectrum students experience mathematical learning in a different manner than their straight peers and in relation to other Queer spectrum identities. Queer-spectrum students with a sexualized identity perceive that mathematics environments are not normative places to be a part of the community, resulting in negative dispositions, reduced interactions, and lower academic success. The impact of sexual identity, although small, contributes to negative indicators of positive mathematical experiences and suggests that the normative discourses in mathematics that are identity neutral, heteronormative, and aligned with binary thinking are differentially impacting Queer-spectrum students.

## Introduction

There has been a growing effort within Science, Technology, Engineering, and Mathematics (STEM) education to broaden participation, address equitable outcomes, and promote inclusive learning environments along an array of various social identities. Given the need for increasing the number of STEM graduates, recent research has focused on the underrepresentation of certain student social identities in STEM (e.g., women, students of color, and students with a disability). Students with these identities are often disproportionately impacted by systemic barriers from obtaining successful educational outcomes [1–3], such as microaggressions [4,5], lack of access to career counselors or advanced STEM courses [6,7], and inaccessible learning spaces [8]. These barriers contribute to higher rates of switching out of STEM majors [9] or leaving college altogether. Efforts to increase outcomes for underrepresented groups have resulted in a myriad of research that addresses structural inequalities (e.g., access to content), psychological factors (e.g., stereotype threat, bias), teaching practices (e.g., research opportunities, active learning), and positive identity formation and belonging within mathematics.

At the same time, educational research, institutional programs, and policies to support students with a minoritized sexual identity in undergraduate mathematics environments remain largely underdeveloped and undertheorized. By minoritized sexual identity, which we henceforth refer to as Queer-spectrum, we mean students who identify as Lesbian, Gay, Bisexual, Pansexual, Questioning, Asexual, or in other ways Queer because of their sexual identity [10]. The choice of the term Queer-spectrum is purposeful, as it represents a political statement in terms of reclaiming the transgressive nature of the word Queer, a term which has historically been used to denigrate individuals in society [11,12]. Additionally, the term Queer-spectrum serves as a mechanism to coalesce among the many different Queer identities (e.g., Bisexual, Lesbian, Pansexual) to understand their shared experiences in mathematics while also recognizing that there are differences and fluidity among these identities.

A majority of the research examining the experiences of Queer-spectrum undergraduate students consists of campus climate studies that gauge the overall openness or hostility in higher education encountered by Queer-spectrum students and faculty [13]. Generally speaking, campus climate research paints a chilly and even hostile environment for Queer-spectrum students, faculty, and staff. Queer-spectrum college students are more likely to describe their college campus as hostile and rate their campus environment less positively than their straight peers [14]. Queer-spectrum students also report high rates of harassment (42% of all Lesbian, Gay, and Bisexual students and 55% of Transgender students) and fear getting a bad grade because of a hostile classroom environment (11% of Lesbian, Gay, and Bisexual students and 15% of Transgender students) [13]. Furthermore, there is growing evidence to highlight that the chilly and exclusionary climate in higher education is further heightened within STEM environments [15–18]. This hostile environment may limit Queer-spectrum college students’ ability to reach their academic potential, as students report that a positive campus climate contributes to their overall academic success [14].

There are, however, emerging efforts within STEM fields to address the inclusion of Queer-spectrum individuals. For example, some professional societies are forming committees to support Queer-spectrum people and inclusive environments in STEM fields (e.g., American Astronomical Society Working Group on LGBTIQ Equity, American Society for Engineering Education’s LGBTQ+ Advocacy in STEM Virtual Community of Practice). Additionally, there have been calls to include sexual identity questions on surveys from the National Science Foundation in order to account for Queer-spectrum people in the analysis of academic and STEM outcomes [19,20]. Collecting such data can help promote visibility and further support research on Queer-spectrum students’ experiences.

The study presented here is part of a larger transformative mixed-methods study examining the experiences, resources, and normative discourses within mathematics for Queer-spectrum students [21]. In this paper, we draw on large-scale quantitative data from students enrolled in undergraduate mathematics courses across the United States. As such, this study presents one of the first and only broad portraits of undergraduate mathematics courses as they are experienced and reported on by Queer-spectrum students. Such an analysis in the discipline of mathematics and introductory math courses (Precalculus, Calculus 1, and Calculus 2) is crucial for understanding equity for Queer-spectrum students, as these courses and the content they contain often serve as gatekeepers to careers and identity formation within STEM fields [1,22]. In that aim, we are guided by the following research question: *Are there differences in reported access of mathematical learning environments related to sexual identity*? *Is sexual identity related to students’ mathematical instructional experiences*?

## Literature review: Queer issues in STEM and mathematics

Evidence suggests that marginalization due to sexuality might be felt more acutely within STEM-related disciplines. In a qualitative study of departmental climate, Queer-spectrum faculty in science and engineering described “overt hostility, a sense of invisibility, interpersonal discomfort, and pressure to ‘cover’ one’s sexuality” [23]. Similar trends are reported from Queer-spectrum students in STEM who are hesitant to disclose (come out about) their sexual identity [24] or downplay its importance [25]. One might reveal their Queer identity to others, which can promote a sense of self-integration and personal empowerment [26,27]; however, the ability and decision to reveal one’s Queer identity is often multifaceted, situational, and not always desired. For instance, Toynton [28] put forth the notion of Queer identity in STEM as the “invisible other,” such that being Queer-spectrum is an experience of being the “other” and yet invisible if wished. The sometimes-invisible nature of Queer identity provides agency to reveal one’s identity while at the same time requiring ongoing decision-making to determine whether and how to disclose this identity which can create a cognitive burden for Queer-spectrum students in STEM environments. For example, research indicates that having to navigate coming out in educational spaces creates more emotional and psychological work for Queer-spectrum students than straight students and often results in daily decisions about revealing their sexuality in the classroom [28–31].

Hughes [15] conducted one of the first longitudinal studies of Queer-spectrum students’ retention in STEM fields at the undergraduate level. Drawing on multilevel regression with student survey data, Hughes documented that Queer-spectrum students were 7% more likely to switch from a STEM major to a non-STEM major compared to their straight peers. Switching rates increased to a 10 percentage-point difference when controlling for retention factors such as undergraduate research and STEM identity [15]. Research on factors related to STEM switching is often associated with power structures that impact a student’s perceptions of feeling "fit and community" within the environment [32] or are negatively correlated with reported experiences of discrimination [33].

Focusing specifically on mathematics, Fischer [34] explored through qualitative methods how six undergraduate Queer-spectrum students affiliated with a local LGBT center integrated their Queer identity with their mathematical identity. Fischer documented that having support for one’s Queer identity at school was found to relate to possessing a stronger mathematical identity. For example, having a Gay mathematics teacher as a role model, receiving tutoring support at the LGBT center, and having Gay-Straight alliances, supported students’ success and engagement in mathematics. Alternatively, students who spoke of feeling sexualized in mathematics classrooms and not wanting to ask questions for fear of being labeled as that “Gay kid asking questions” presented challenges for engaging fully with their mathematical identity.

The relatively few quantitative studies of Queer-spectrum students in relation to math have mostly examined outcomes for non-heterosexual high school students using the National Longitudinal Study of Adolescent Health data, often resulting in mixed findings. For instance, Gottfried and colleagues [35] claimed that status as a sexual minority does not contribute any additional explanatory power in predicting advanced course-taking patterns in mathematics. In contrast, Russell and colleagues [36] analysis of the same data supports the notion that same-sex attracted youth do have lower academic outcomes and that relationships with teachers may play a leading role in explaining this difference. In a separate analysis using the same data set, Pearson, Muller, and Wilkinson [37] showed that same-sex attracted youth do worse on measures of academic achievement, higher on measures of emotional distress and substance abuse, are less socially integrated with their schools and teachers, and have lower expectations for attending college. Pearson and colleagues showed using logistic regression that, even when controlling for feelings of attachment and engagement in school, same-sex attracted boys are approximately 47 percent less likely to complete algebra II and 41 percent less likely to complete chemistry compared to their opposite-sex attracted peers [37]. Interestingly, this same trend was not present in course taking patterns for foreign language, suggesting that something is “unique about mathematics and science that makes them more intimidating than other subjects” [37]. The study presented here helps address a clear need in the literature given the sparse number of studies in this domain, and the need to gain more nuanced understanding of Queer-spectrum students experiences in undergraduate mathematics beyond performance and retention measures.

## Theoretical perspective

This study draws on Queer theory to conceptualize, analyze and report on findings related to Queer-spectrum student experience in undergraduate mathematics. Queer theory seeks to account for how normative assumptions regarding sex, sexuality, gender, and gender identity have been shaped by institutional structures. Queer theory is often used to deconstruct dominant theories of identity, position identity as culturally and historically situated, and recognize the fluid nature of identity throughout a person’s lifetime [38]. Queer theory explores the phenomenon of otherness through interrogating anything that comes between normative and deviant activities [39]. One of the principal aims of Queer theory is to challenge what is considered normal and to offer alternative ways of thinking and performing in the world [40]. Butler [38] contends that through social institutions, we become naturalized to normative assumptions prescribed by the *heterosexual matrix*, which positions gender (man, woman) and sexual identity (straight, Gay, Bisexual) as a set of finite, discrete categories. McWilliams and Penuel [40] contend that all human activity is mediated through the heterosexual matrix, making it either difficult to see things that fall outside one of the six entries in the matrix, or making such things deviant and a target for obliteration. For example, individuals who identify as cisgender men may transgress normative assumptions of gender by performing in drag and wearing make-up and dresses in certain contexts. These individuals may fall outside one of the six cells and, thus, are either not acknowledged as being fully cisgender men or may even be targeted as the victims of violence for transgressing against the heterosexual matrix. Queer theory attempts to expose the tensions that reside within the heterosexual matrix by decentering identity as a fixed category. Instead, it draws on post-structural theories that view identity as performative [38]. By performative, we mean that social constructs and identity are based on repeated imitations of social norms and not internal qualities. In this study, we avoided placing rigid boundaries around categories like Queer-spectrum and instead opted to let participants decide whether those words or others applied to them or not. Viewing identity as performative and resisting binary categorization allows Queer theorists to account for the ways that identity may be fluid and changing, both situationally in different environments and temporally throughout the lifespan of an individual [41,42].

Because Queer theory seeks to deconstruct dominant theories of identity and resist normative categorization, there is an inherent tension in using quantitative methods to analyze sexual identity because it often results in a reductionist view of Queerness [43]. We recognize that the analysis presented here, while drawing on notions of queer theory in study design and interpretations, still results in reductionist views of identity and experience. However, there are a growing number of researchers arguing for the use of quantitative methods that more closely align with Queer theory [43–45]. These researchers seek to center Queerness by questioning commonly held methodological beliefs such as neutrality, generalizability, and categorization. Queer theory helps guide this study by (1) coalescing Queer-spectrum participants as a lens in this study to include Lesbian, Gay, Bisexual, Pansexual, Questioning, Asexual, and individuals who are “Queer” because of their exclusion from normative sexual identities; (2) resisting essentializing the experience of all Queer-spectrum students as similar by attending to the varied nature of the self and not drawing binary comparisons between straight and an overall Queer-spectrum student identity; (3) accounting for the ways that Queer identity is performed and can vary across different situational contexts; (4) facilitating the interpretation of our results to highlight how structures and norms may produce results for various students versus viewing the results as essentialized and generalized differences among sexual identities; and (5) acknowledging limitations in our study design since Queer performance is viewed through a heteronormative society as deviant and thus may be regulated by students and others while learning mathematics or reporting such experiences in survey responses.

## Methods

The San Diego State University Institutional Review Board approved this study (#2449100). Written consent was obtained from all participants. The data from this study comes from 20 universities across the United States as a result of two national studies of introductory mathematics courses, Progress through Calculus (PtC) and Student Engagement in Mathematics through an Institutional Network for Active Learning (SEMINAL). All the universities offer at least a master’s degree in mathematics and represent various institutional types (private/public, HBCU/HSI/PWI, technical, medium/large undergraduate enrollment). Universities from the PtC project were selected to represent various features of successful calculus programs. Universities from the SEMINAL project were selected based on departmental efforts to implement active learning in introductory mathematics courses. As part of the projects, we developed the Student Post-Secondary Instructional Practices—Mathematics (SPIPS-M) survey [46,47] and administered it during the Fall 2017, Spring 2018, and Fall 2018 academic terms. The SPIPS-M is a comprehensive survey instrument designed to measure the instructional environment and experiences of students in mathematics courses.

We identified through prior factors analysis [48] four scales related to instructional practices: *Instructor interactions*, *Peer interactions*, *Community & participation*, *and Math engagement*. Items on the scale range from 1 (does not occur) to 5 (very descriptive). *Math affect* is a 4-item scale containing items related to confidence, interest, enjoyment, and sense of ability in mathematics that range from 1 (strongly disagree) to 6 (strongly agree). The *Expected grade* item on the survey measures what grade students anticipate receiving in the course measured when the survey was administered roughly three-quarters of the way through the academic term. Although this is not the final course grade, it serves as a proxy for their course performance. Prior research indicates that generally, students in math and science courses are able to predict their course performance 73% of the time [49]; however, this item should also be interpreted with caution as expected grades can be influenced by social markers (e.g., gender, international status). At the same time, we include *Expected grade* as it is an important factor in how students experience their mathematical learning environments, and it is often a compelling metric for researchers and policymakers to identify opportunity gaps. Response options for *Expected grade* were coded as 1 (F), 2 (D), 3 (C, C+ or C-), 4 (B, B+ or B-), 5 (A, A+ or A-) and the choice of “other” was removed for analysis. The inter-rater reliability metrics Cronbach alpha and Cohen’s Kappa were calculated for the scales. The inter-rater reliability scores meet the moderate acceptability threshold (Cronbach Alpha >.70, and Cohen’s Kappa >.41) to treat these items as a scale [50]. Each of the relevant measures is outlined in Table 1.

**Table 1:** Scale Measures and Interrater reliability scores of math instructional experiences.

Measure Name	Description	Sample item	Items	Cronbach Alpha	Cohen’s Kappa
*Instructor interactions*	Students report activities consistent with sharing thinking with the instructor and receiving feedback	I am asked to respond to questions during class time	3	.70	.41
*Peer interactions*	Students report activities consistent with collaborating to discuss and critique mathematics. They share ideas in small groups as well as with the class and collaborate to support each other	I talk with other students about course topics during class	7	.86	.43
*Community & participation*	Students report that a wide variety of students are included in the mathematical discussions in the course and that there is a sense of community among the students in the course	A wide range of students respond to the instructor’s questions in class	3	.82	.61
*Math Engagement*	Students report activities consistent with meaningful engagement with mathematics involves more than memorization, but internalization, connections across content and disciplines, multiple representations, responsive environment; course structure promotes engagement	The class activities connect course content to my life and future work	9	.89	.43
*Math affect*	A measure of affect towards mathematics which includes confidence, interest, enjoyment, and sense of ability	Now, I am interested in mathematics	4	.89	.62
*Expected Grade*	A measure of the student’s anticipated course grade	What grade do you expect to get?	1	-	-

Drawing from Queer theory, which seeks to deconstruct how identity and experiences vary situationally, we identified all items on the survey related to the different math learning environments outlined in Table 2. As such, we included single-item questions for examinations related to external-classroom attendance, mostly related to help-seeking *(Instructor*, *Tutoring*, and *Peers)* and course attendance (*Regular class* and *Breakout/recitation*). External-classroom attendance response options were Likert-type scale: 1 (does not occur), 2 (minimally), 3 (somewhat), 4 (mostly), and 5 (very descriptive). Response options for *Course attendance* were categorical and included almost never, occasionally, frequently, and more than half.

**Table 2:** Items related to math learning environments.

Item	Item Prompts
**External-Classroom attendance**	
*Instructor*	I see my instructor(s) outside of class for help
*Tutoring*	I attend tutoring sessions outside of class time
*Peers*	I work with peers outside of class on math problems
**Course attendance**	
*Regular class*	Roughly how often have you missed class meetings for the course?
*Breakout/Recitation*	Roughly how often have you missed recitation meetings for the course?

We analyzed the data using descriptive statistics, Chi-square test of Independence, MANOVA, and post hoc tests. The Chi-square test for independence was used to measure the relationship between sexual identity and course attendance. MANOVA was used to measure the association between sexual identity and the dependent continuous variables measuring instructional experiences and another for external-classroom attendance. The following conditions were confirmed prior to running the MANOVA: there were not any relationships between the observations in each group and between groups, there were no extreme (multivariate) outliers in the dependent variables, multivariate normality was met, and multicollinearity was not present, linearity between the dependent variables, and homogeneity of variance-covariance matrices and variances were present.

One of the first challenges in addressing this research goal is determining how to account for Queer-spectrum identity using quantitative methods. One of the first steps towards this alignment is using sexual identity categories beyond Gay and Lesbian (e.g., Bisexual, Asexual), which can help center Queer theory on the experiences of Bisexual and Asexual individuals who represent a large portion of Queer-spectrum individuals in society. Another way in which scholars have sought to align quantitative methods with Queer theory is through survey design and the creation of indicators that deconstruct normative categorization impulses [43,45]. For example, Browne [45] used grouping techniques with large enough samples to create new Queer identity categories based on how individuals responded to questions about sexual identity, sexual attraction, and relationship status. Using similar grouping techniques, we sought to resist normative categorization by allowing survey respondents to select multiple options and included write-in choices. The format of the sexual identity question was “(Select all that apply) Do you consider yourself to be:” and response options were “Asexual, Bisexual, Gay, straight (heterosexual), Lesbian, Queer, Not listed (please specify), and Prefer not to disclose.” A total of 25,785 student survey responses were received. We conducted data cleaning based on recommendations from Cimpian [51] in order to avoid classification errors and bias when researching sexualized identities. We removed malicious responses (*n* = 197) through screening techniques which included responses checking all items to a demographic question or providing a response in the write-in options that were deemed frivolous or malicious (e.g., listing “Apache helicopter” for gender).

## Results

### Accounting for Queer-spectrum identities

A total of 24,327 students responded to the sexual identity question on the survey. There were 107 open-ended responses which we analyzed and then categorized. The most common write-in responses included pansexual (*n* = 56), questioning (*n* = 11), demisexual (*n* = 8), or the responses provided were categorized as an existing response option (*n* = 29). Since multiple options could be selected when responding to the sexual identity question, we created a categorical variable to indicate the students’ desired responses. For instance, if a student selected both straight and Bisexual, a categorical variable called Straight-Bisexual was assigned to this student. In the first round of classification, we considered all categorical variables and counted each possible response. We then conducted an iterative binning process informed by Queer theory and response counts to combine categories when appropriate [40,43]. The first round of binning resulted in 13 sexual identities, see [21]. We then conducted a second iteration of binning to combine categories with smaller response counts in order to have categories that are communicative and interpretable for the reader and large enough to allow for statistical inferences. A new category of Queer+ (read Queer plus) was used to indicate students who indicated Pansexual or Demisexual (*n* = 64), Queer (*n* = 94), or Multiple Queer-Spectrum options (e.g., Gay and Bisexual, Asexual and Lesbian) (*n* = 119). A new category of Straight+ (read Straight plus) was used to combine students indicating Straight-Asexual (*n* = 77), Straight-Bisexual (*n* = 53), Straight-Multiple (*n* = 22) and Questioning (*n* = 11). Considering these students to be Queer-spectrum is informed by our theoretical perspective of Queer theory, which interrogates categorical essentialization and is consistent with research suggesting that some adults, while uncomfortable indicating Bisexual still report same-sex attractions [52]. A summary of the categories, along with response counts and percentages, is presented in Table 3. There were 2,454 Queer-spectrum student responses across 898 classrooms. Queer-spectrum students account for 10.0% (*n* = 2,454) of the total student responses to the sexual identity question (*n* = 24,327). The prevalence of Queer-spectrum students in this study is an important finding, as it provides evidence to instructors of the presence and likelihood of Queer-spectrum students in introductory math courses.

**Table 3:** Sexual Identity categories with response counts and percentages.

Category	Sexual identity	Count	Percentage
Queer-spectrum	Asexual	633	2.6%
	Bisexual	932	3.8%
	Gay	303	1.2%
	Lesbian	146	0.6%
	Queer+	277	1.1%
	Straight+	163	0.7%
Straight	Straight	20855	85.7%
Not disclose	Not disclose	1018	4.2%

### Queer-spectrum students and math learning environments

**Fig 1:**
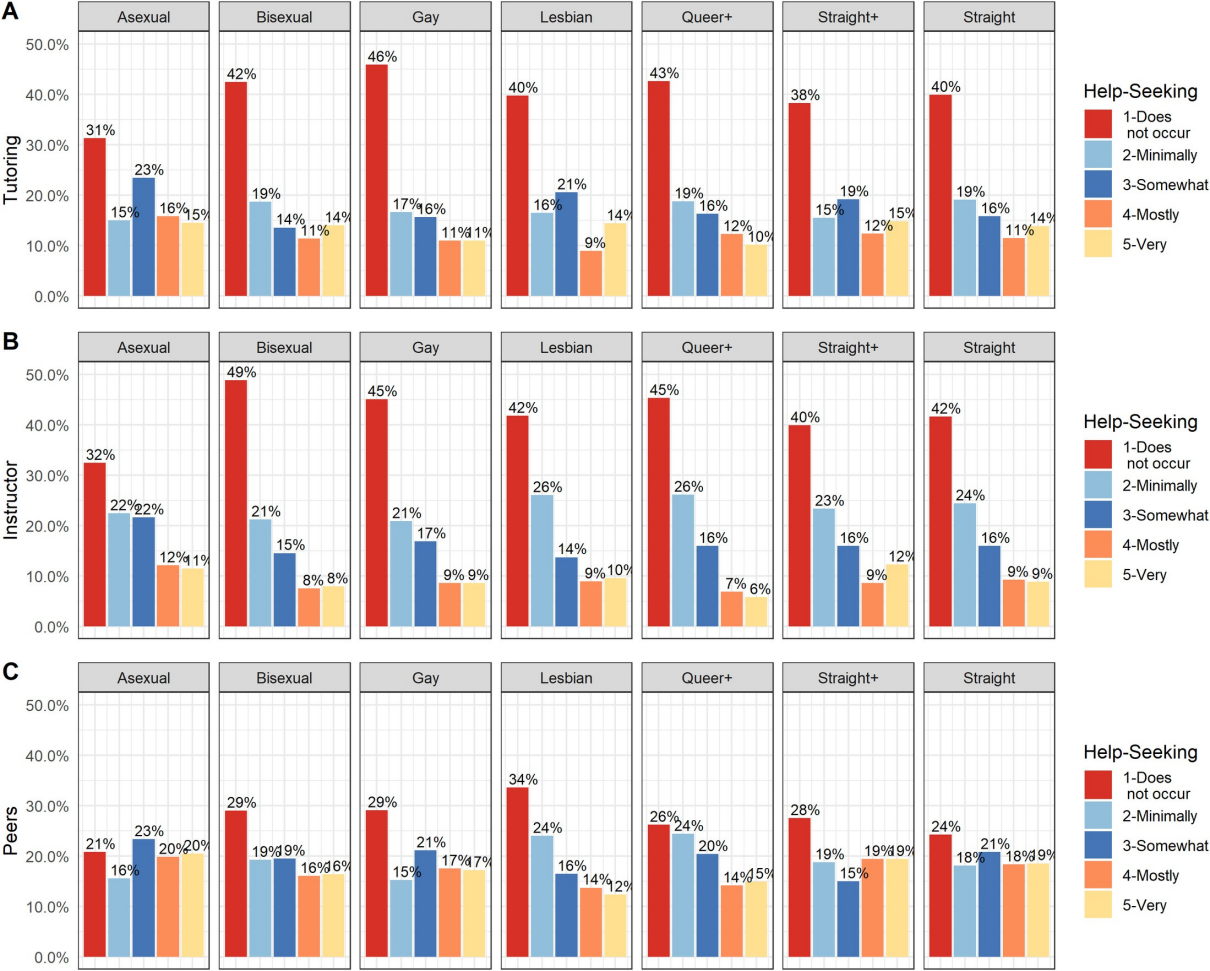
Boxplot with mean of external-classroom attendance across sexual identities in math learning environments outside the classroom, which include (a) instructor, (b) tutoring, and (c) peers.

A one-way multivariate analysis of the variance (MANOVA) was conducted to test for differences between sexual identity and external-classroom attendance. A statistically significant MANOVA effect was obtained (*Wilk’ s lambda* = 0.996, *F*(6,23152) = 4.903; p < .001, *η*^2^ = .0013). Using the Bonferroni method, each ANOVA was tested at a .016 (.05/3) alpha level. Results demonstrated that there was sufficient evidence to determine a significant effect of sexual identity on all measures of external-classroom attendance (see Table 4)–*Instructor* (*F*(6,23152) = 7.98; *p* < .001, *η*^2^ = .0021), *Tutoring* (*F*(6,23152) = 5.03; *p* < .001, *η*^2^ = .0013) and *Peers* (*F*(6,23152) = 6.48; *p* < .001, *η*^2^ = .0017). A graphical visualization of the data for external-course attendance by sexual identity is presented in Fig 1.

**Table 4:** Means, standard deviation, and one-way manova for external-course attendance, math instructional experiences, and sexual identity.

Measure	Asexual		Bisexual		Gay		Lesbian		Queer+		Straight+		Straight		*F*	*p*	η^2^
	*M*	*SD*	*M*	*SD*	*M*	*SD*	*M*	*SD*	*M*	*SD*	*M*	*SD*	*M*	*SD*			
**External-course attendance**																	
Instructor	2.48	1.35	2.05	1.28	2.15	1.31	2.18	1.32	2.02	1.19	2.30	1.39	2.19	1.50	7.98	< .001	0.0021
Tutoring	2.67	1.43	2.36	1.46	2.25	1.41	2.42	1.45	2.29	1.38	2.50	1.47	2.40	1.39	5.03	< .001	0.0013
Peers	3.04	1.42	2.71	1.44	2.79	1.46	2.47	1.40	2.67	1.39	2.84	1.50	2.89	1.44	6.48	< .001	0.0017
**Instructional Experiences**																	
Instructor interactions	2.90	1.12	2.57	1.09	2.63	1.13	2.71	1.11	2.55	1.02	2.70	1.00	2.69	1.07	5.52	< .001	0.0014
Peer interactions	2.97	1.01	2.70	1.03	2.72	1.08	2.82	1.05	2.60	1.03	2.78	0.94	2.78	1.00	5.23	< .001	0.0014
Community & participation	3.15	1.05	2.91	1.07	2.86	1.08	3.05	1.12	2.81	0.95	2.93	1.02	3.04	1.04	6.91	< .001	0.0019
Math engagement	3.54	0.86	3.42	0.91	3.53	0.94	3.49	0.99	3.51	0.80	3.50	0.82	3.55	0.87	3.50	0.002	0.0009
Math affect	4.30	1.30	4.03	1.33	4.16	1.33	4.12	1.30	4.19	1.31	4.17	1.30	4.32	1.24	9.07	< .001	0.0024
Expected Grade	3.83	0.98	3.92	0.92	3.99	0.93	3.82	0.93	3.96	0.92	3.87	0.98	3.98	0.89	4.59	< .001	0.0012

A pairwise comparison using the Games-Howell post hoc tests was computed (see Table 5) for external-classroom attendance items and sexual identity. There were differences in *Instructor* environment with higher levels of attendance for Asexual (M = 2.48; SD = 1.35) students compared to Gay (M = 2.15; SD = 1.31), Queer+ (M = 2.02; SD = 1.19), Bisexual (M = 2.05; SD = 1.28) and straight (M = 2.19; SD = 1.50) students and higher levels for straight (M = 2.19; SD = 1.50) students compared to Bisexual (M = 2.05; SD = 1.28) students. For *Tutoring*, there were significant differences with higher levels for Asexual (M = 2.67; SD = 1.43) students compared to Gay (M = 2.25 SD = 1.41), Queer+ (M = 2.29; SD = 1.38), Bisexual (M = 2.36; SD = 1.46), and straight (M = 2.40; SD = 1.39) students. For *Peers*, there were significant differences with higher levels for Asexual (M = 3.04; SD = 1.42) students compared to Lesbian (M = 2.47; SD = 1.40), Queer+ (M = 2.67; SD = 1.39), and Bisexual (M = 2.71; SD = 1.44) students, and higher levels for straight (M = 2.89; SD = 1.44) students compared to Bisexual (M = 2.71; SD = 1.44) and Lesbian (M = 2.47; SD = 1.40) students.

**Table 5:** Significant post hoc test of external-classroom attendance in math learning environments and sexuality.

Measure	Pairwise comparison (Games-Howell)		Mean difference	95% CI lower	95% CI upper	Adjusted p-value	Cohen’s *d*
Instructor	Asexual	Queer+	-0.455	-0.720	-0.189	< .001	0.35
	Asexual	Bisexual	-0.425	-0.628	-0.223	< .001	0.32
	Asexual	Gay	-0.330	-0.604	-0.055	.007	0.24
	Asexual	Straight	-0.280	-0.442	-0.117	< .001	0.22
	Bisexual	Straight	0.146	0.019	0.273	.013	0.11
Tutoring	Asexual	Gay	-0.437	-0.731	-0.144	< .001	0.30
	Asexual	Queer+	-0.399	-0.696	-0.101	.002	0.27
	Asexual	Bisexual	-0.317	-0.537	-0.096	< .001	0.21
	Asexual	Straight	-0.272	-0.443	-0.100	< .001	0.18
Peers	Asexual	Lesbian	-0.556	-0.939	-0.173	< .001	0.40
	Asexual	Queer+	-0.356	-0.655	-0.057	.008	0.26
	Asexual	Bisexual	-0.317	-0.535	-0.099	< .001	0.22
	Bisexual	Straight	0.176	0.033	0.319	.006	0.12
	Lesbian	Straight	0.414	0.068	0.761	.008	0.29

**Fig 2:**
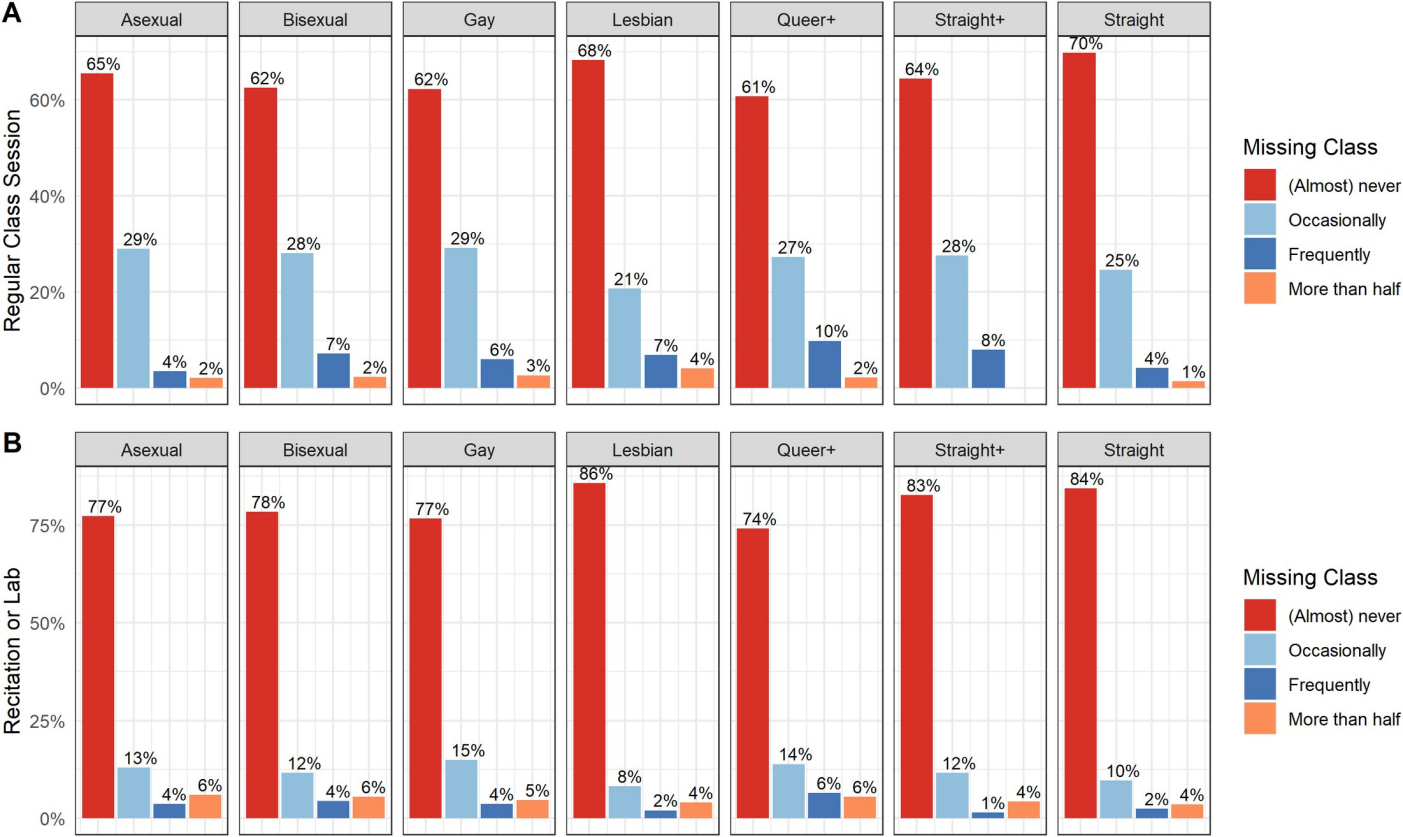
Histogram of course attendance in both Regular class and Breakout/Recitation across sexual identities.

For each of the Course attendance items, a histogram of response options across sexual identities was created and is presented in Fig 2. We performed a Chi-square test of independence to examine the relationship between sexual identity and course attendance (both *Regular class* and *Breakout/Recitation*). The relation between *Regular class* and sexual identity was significant, *χ*^2^ (18, *N* = 23115) = 90.03; *p* < .001, *Cramer’ s v* = .04. The relation between *Breakout/Recitation* and sexual identity was significant χ^2^ (18, *N* = 9065) = 34.55; *p* = .011,*Cramer’ s v* = .04. Straight and Lesbian students reported the lowest levels of missing *Regular class* meetings and *Breakout/recitation*. Queer+ students reported the highest levels of missing either *Regular class* meetings or *Breakout/recitation*.

Interpreting these results, within *Peer* environments, Lesbian students are less likely to access these spaces, and within *Instructor* environments, Bisexual students are less likely to access those spaces. Asexual students reported the highest levels of attending any external-classroom environments. Overall, students were more likely to report attending *Peer* environments compared to *Tutoring* or *Instructor* environments. These findings highlight how various instructional environments outside the classroom may be exclusionary or less accessible for certain students and how they are experienced differently among Queer-spectrum students. Furthermore, we see that within classroom environments, both *Regular class* sessions and *Breakout/recitation*, there was a relationship in attendance based on sexual identity. There are differences in how likely students are to access various learning environments in both formalized learning contexts such as classrooms, instructor offices, and tutoring sessions, as well as informal learning environments like working with peers. Further analysis is needed to examine the structures and contexts that are resulting in these various patterns but highlight how future studies must attend to the environments in which learning is occurring for Queer-spectrum students.

### Queer-spectrum students and math instructional experiences

**Fig 3:**
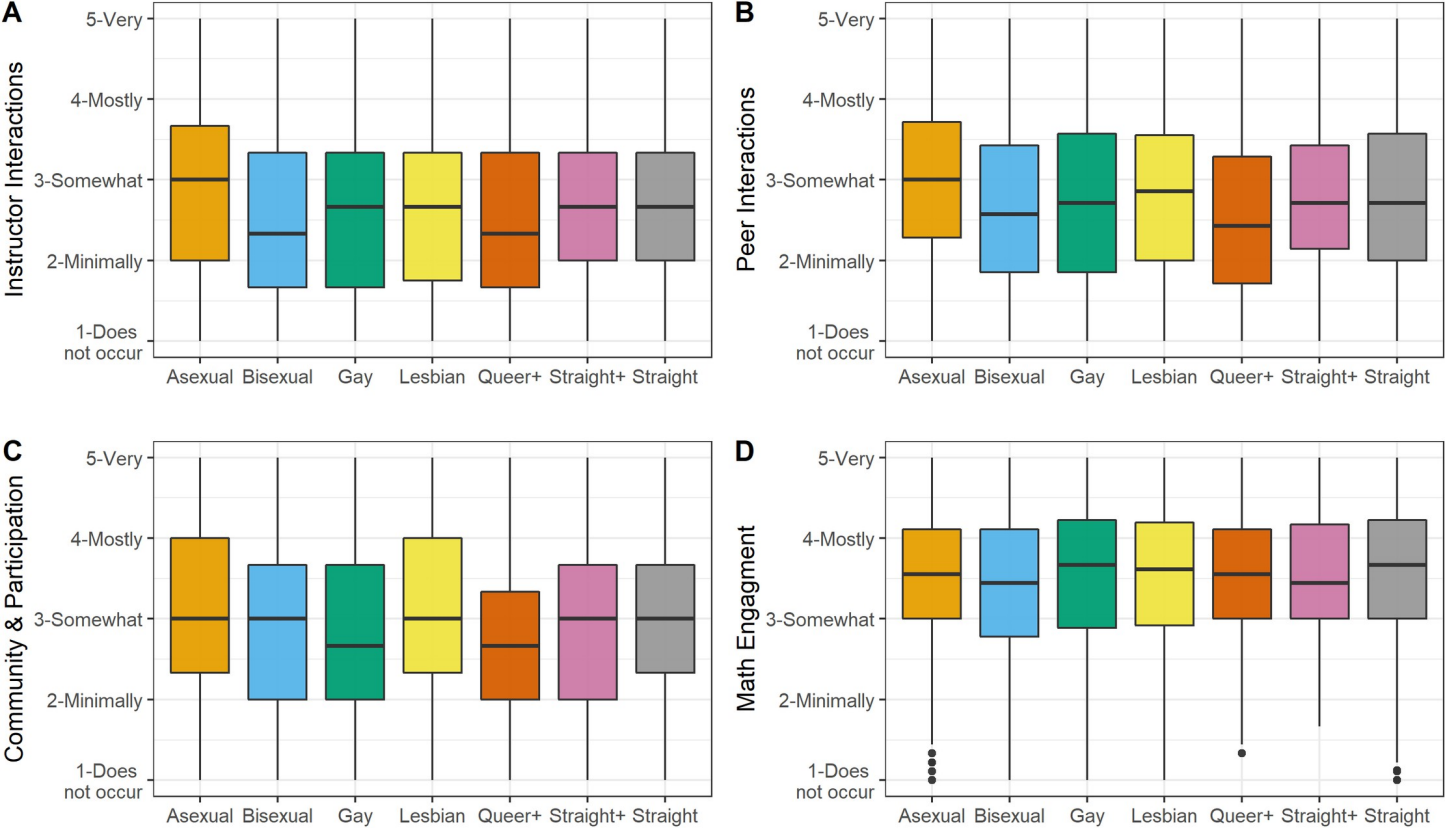
Boxplot with mean of Instructor interactions (A), Peer Interactions (B), Community and Participation (C), and Math Engagement (D) across sexual identity.

**Fig 4:**
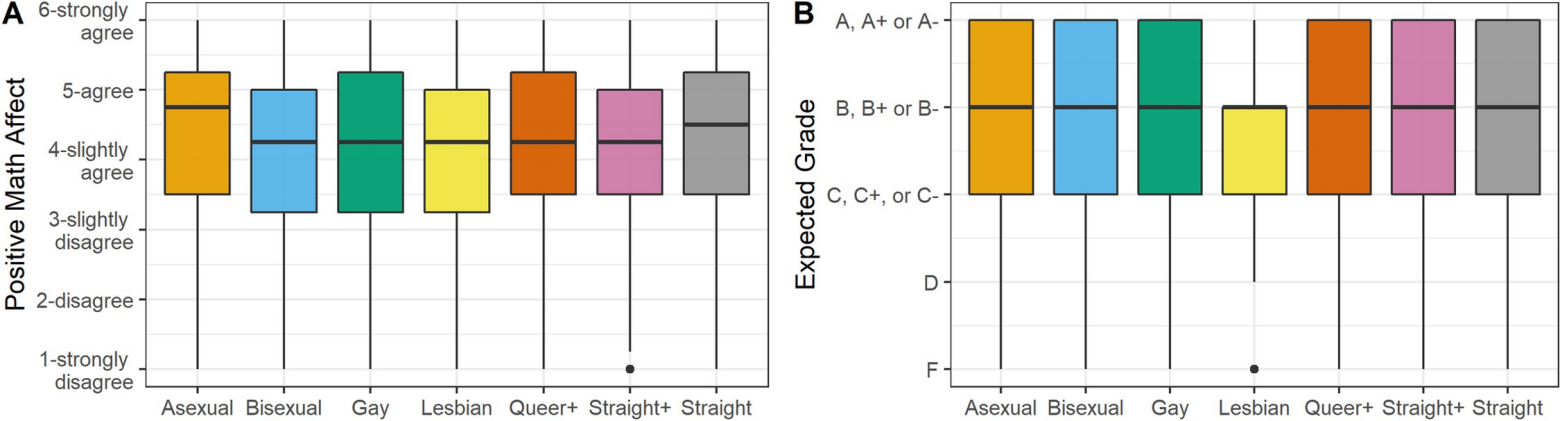
Boxplot with mean of Math Affect (A) and Expected Grade (B) across sexual identity.

A one-way multivariate analysis of the variance (MANOVA) was conducted to test for differences between sexual identity and mathematical instructional experiences. A statistically significant MANOVA effect was obtained (*Wilk’ s lambda* = 0.992,*F*(6,22,337) = 4.709; *p* < .001, *η*^2^ = .0013). Using the Bonferroni method, each ANOVA was tested at a .007 (.05/6) alpha level. Results demonstrated that there was sufficient evidence to determine a significant effect of sexual identity on all measures of mathematical instructional experiences (see Table 4)—*Instructor interactions* (*F*(6,22337) = 5.52; *p* < .001, *η*^2^ = .0014), *Peer interactions* (*F*(6,22337) = 5.23; *p* < .001, *η*^2^ = .0014), *Community & participation* (*F*(6,22337) = 6.91; *p* < .001, *η*^2^ = .0019), *Math engagement* (*F*(6,22337) = 3.50; *p* = .002, *η*^2^ = .0009), *Math affect* (*F*(6,22337) = 9.07; *p* < .001, *η*^2^ = .0024), and *Expected grade* (*F*(6,22337) = 4.59; *p* < .001, *η*^2^ = .0012). A graphical visualization of the data for mathematical instructional experiences and sexual identity is presented in Figs 3 and 4.

A pairwise comparison using the Games-Howell post hoc tests was computed (see Table 6). For *Instructor interactions* there were statistically significant differences with Asexual (M = 2.90; SD = 1.12) students reporting higher levels compared to Gay (M = 2.63; SD = 1.13), Queer+ (M = 2.55; SD = 1.02), Bisexual (M = 2.57; SD = 1.09) and straight (M = 2.69; SD = 1.07) students and higher levels for straight (M = 2.69; SD = 1.07) students compared to Bisexual (M = 2.57; SD = 1.09) students. For *Peer interactions*, a difference in mean score was significant with higher levels for Asexual students (*M* = 2.97, *SD* = 1.01) compared to Gay (M = 2.72; SD = 1.08), Queer+ (M = 2.60; SD = 1.03), Bisexual (M = 2.70; SD = 1.03) and straight (M = 2.78; SD = 1.00) students. For *Community & participation*, responses for Asexual students (*M* = 3.15 *SD* = 1.05) and straight students (M = 3.04 SD = 1.04) were pair-wise statistically significantly compared to Gay (M = 2.86; SD = 1.08), Queer+ (M = 2.81; SD = 0.95), and Bisexual (M = 2.91; SD = 1.07) students. For *Math engagement*, a statistically significant difference in mean score for Bisexual students (*M* = 3.42, *SD* = 0.91) was lower compared to straight (M = 3.55; SD = 0.87) students.

**Table 6:** Significant post hoc test of math instructional experiences and sexuality.

Measure	Pairwise comparison (Games-Howell)		Mean difference	95% CI lower	95% CI upper	Adjusted p-value	Cohen’s *d*
Instructor interactions	Asexual	Gay	-0.262	-0.495	-0.029	0.016	0.23
	Asexual	Queer+	-0.344	-0.569	-0.120	0.000	0.32
	Asexual	Straight	-0.208	-0.342	-0.074	0.000	0.19
	Asexual	Bisexual	-0.327	-0.496	-0.158	0.000	0.30
	Bisexual	Straight	0.119	0.011	0.227	0.020	0.11
Peer interactions	Asexual	Gay	-0.250	-0.468	-0.031	0.014	0.24
	Asexual	Bisexual	-0.274	-0.429	-0.119	0.000	0.26
	Asexual	Queer+	-0.369	-0.587	-0.151	0.000	0.36
	Asexual	Straight	-0.190	-0.310	-0.069	0.000	0.19
Community & participation	Asexual	Gay	-0.290	-0.511	-0.069	0.002	0.27
	Asexual	Bisexual	-0.241	-0.402	-0.081	0.000	0.23
	Asexual	Queer+	-0.343	-0.553	-0.134	0.000	0.34
	Bisexual	Straight	0.137	0.031	0.243	0.003	0.13
	Gay	Straight	0.185	0.001	0.370	0.049	0.18
	Queer+	Straight	0.239	0.068	0.410	0.001	0.23
Math Engagement	Bisexual	Straight	0.128	0.154	0.418	0.001	0.15
Math affect	Asexual	Bisexual	-0.268	-0.470	-0.065	0.002	0.20
	Bisexual	Straight	0.286	0.038	0.219	0.000	0.23
Expected Grade	Asexual	Straight	0.145	0.028	0.262	0.005	0.16

For *Math affect*, a statistically significant difference in mean score for Bisexual students (*M* = 4.03, *SD* = 1.33) was lower compared to Asexual (M = 4.30; SD = 1.30) and straight (M = 4.32; SD = 1.24) students. For *Expected grade*, a difference in mean score for Asexual students (*M* = 3.83, *SD* = 0.98) was statistically lower compared to straight (M = 3.98; SD = 0.89) students.

Looking across these results, we see that Asexual students reported higher levels of *Instructor interactions*, *Peer interactions*, and *Community & Participation* compared to Gay, Bisexual, Queer+, and straight students. Asexual students also report higher levels of *Math Affect* compared to Bisexual students, while at the same time report a lower *Expected grade* compared to straight students. Aside from Asexual students, Bisexual students had the most significant pairwise differences, reporting lower levels of *Instructor Interactions*, *Community & Participation*, *Math Engagement*, and *Math Affect*. These results seem to indicate that the mathematical environments are more conducive to students who identify as Asexual and less conducive for students who identify as Bisexual.

## Discussion

Looking across all significant findings, the relationship between sexual identity and math learning experiences and math environments was largely attributed to reported differences from Asexual students. Asexual students reported high levels of *Instructor interactions*, *Peer interactions*, *Community & participation*, and *Math affect* and were the most likely to access learning environments external to the course. These findings stand in contrast to existing research that suggests Asexual students report lower levels of school belonging [53] and experience a denial of epistemic agency from others (e.g., denying the existence of Asexuality) [54]. Asexuality, which is characterized by a lack of sexual attraction toward any gender, may be an identity that aligns with the normative discourses in mathematics that are identity neutral and assumed heteronormative, meaning that issues of identity are not relevant to the pursuit of mathematics [55,56]. Such a finding would be consistent with data that suggests Asexual students report less victimization due in part to the assumed invisibility of Asexual identity [53].

Given the marked differences for Asexual individuals, there is a need for future research on Queer-spectrum identities to differentiate between sexualized Queer-spectrum students (Bisexual, Gay, Lesbian, Queer, Straight+) and Asexualized Queer-spectrum students (Asexual). Combing all Queer-spectrum students under one category can mitigate differences and result in the appearance of null findings when examining the experiences of Queer-spectrum students in STEM. Taking such an approach to differentiate between sexualized and Asexualized Queer-spectrum students also prevents researchers from disregarding Asexual identity from data analysis and thus erasing the experiences of Asexual students. For instance, although our analysis revealed greater reports of interactions and a sense of community for Asexual students, their positive experiences in classroom interactions did not contribute to their academic success in the course as measured by *Expected grade*, which warrants further investigation.

The result with the largest effect size was related to *Math Affect*. The differences in *Math Affect*, given that this measure is an internalized sense of students’ relationship with mathematics (e.g., one’s mathematical identity), are important to understand in order to support Queer-spectrum students’ mathematical identity development [34]. The findings here suggest that there are factors within introductory math courses that inhibit the development of positive math affect for Bisexual students while promoting those for Asexual and straight students. Furthermore, Bisexual students reported lower levels of *Instructor Interactions*, *Community & Participation*, *and Math Engagement* and were less likely to access Instructor or Peer learning environments compared to straight students. These findings suggest that there is something occurring within the interactional patterns of mathematics classrooms that disengages or positions Bisexual students outside the community. Bisexual identity generally resists binary logics that promote hetero- or homo-normativity. Bisexual identity is often made invisible within Gay/Lesbian and Straight social circles [44]. The pressures of invisibility and alienation within both social circles may contribute to Bisexual students’ sense of not belonging in mathematics classrooms. This effect may be heightened in mathematics classrooms that promote binary oppositions [57].

Somewhat surprising in the research were the limited significant differences for Gay and Lesbian students in relation to other sexualized identities. The existing literature suggests that Gay men often hear homophobic remarks, don’t feel comfortable revealing their STEM identities, and experience challenges in STEM environments [24]. Lesbian students, who have multiple marginalized identities, may experience microaggressions toward gender and sexuality [58]. Yet the results from this study revealed the only statistically significant difference with sexualized identities (e.g., not Asexual students) occurred between Gay and straight students on *Community & participation* in the classroom and between Lesbian and straight students accessing *Peer* environments outside the classroom context. One possible reason that there are not many differences for Gay and Lesbian students is that the survey does not capture how Gay and Lesbian identity impacts students’ experiences in mathematics classrooms. These students may be able to orient themselves to engage in interactions and feel that they belong in mathematics but accomplish this by downplaying their Gay and Lesbian identity [59]. Such results highlight the limitations of the study design that commonly held beliefs about mathematics being identity-neutral might influence homogenous student responses to the survey since heteronormative notions are being valued within math environments.

Although we find significant results in the data that help answer the research questions and contribute to the dearth of research evidence on Queer-spectrum student experiences, it is also important to situate and interrogate the effect size for these reported findings. All of the effect sizes for statistical differences can be interpreted through standard benchmarks as small, with the range of values for η^2^ [.0012-.0024], Cohen’s *d* [.11-.40], and Cramer’s V [.04]. As such, readers should not over-interpret the practical implications of these differences in math classrooms. At the same time, helping to situate these findings, prior research with this same dataset showed the effect sizes for sexual identity as measured in regression models were comparable to differences in gender, race, and first-generation college student status [21]. Given the well-documented disparities for women and students of color in undergraduate mathematics, the findings from this research study should raise similar conversations, programs, and interventions to support Queer-spectrum students. Furthermore, drawing from a Queer theory lens of interrogating statistical assumptions, what level of educational disparities, as measured by effect sizes, is acceptable? Does casting these as small effects minimize their importance and downplay the potential marginalization of Queer-spectrum students? We leave the reader with these questions and inherent tension.

Overall, within introductory math courses, sexual identity did have a significant relationship with accessing learning environments and mathematical learning experiences. These findings suggest that Queer-spectrum students experience mathematical learning opportunities in a different manner than their straight peers and in relation to other Queer-spectrum identities. Some Queer-spectrum students perceive that mathematics environments are not normative places to be a part of the community, resulting in negative dispositions, reduced interactions, and lower academic success. The impact of sexual identity on these measures, although small, contributes to negative indicators of positive mathematical experience. These findings highlight the need to provide support for Queer-spectrum students in mathematics and to systematically alter the culture and environment of undergraduate mathematics to transform them into normative spaces for Queer-spectrum students. Educators must begin to break down binary notions within mathematics and affirm the presence of Queerness as a rightful topic in math courses to ensure that all Queer-spectrum students are included and belong in the endeavor of learning mathematics.
